# Revisiting the Metabolism of Donepezil in Rats Using Non-Targeted Metabolomics and Molecular Networking

**DOI:** 10.3390/pharmaceutics17010115

**Published:** 2025-01-15

**Authors:** Eun-Ji Park, Eui-Hyeon Kim, Ki-Young Kim, Ji-Hyeon Jeon, Im-Sook Song, So-Young Park, Kwang-Hyeon Liu

**Affiliations:** 1 BK21 FOUR KNU Community-Based Intelligent Novel Drug Discovery Education Unit, College of Pharmacy and Research Institute of Pharmaceutical Sciences, Kyungpook National University, Daegu 41566, Republic of Korea; roseej98@naver.com (E.-J.P.); uihyeon1112@naver.com (E.-H.K.); neanic12@naver.com (K.-Y.K.); kei7016@naver.com (J.-H.J.); isssong@knu.ac.kr (I.-S.S.); 2 Mass Spectrometry Based Convergence Research Institute, Kyungpook National University, Daegu 41566, Republic of Korea

**Keywords:** donepezil, mass spectrometry, metabolism, molecular networking, nontargeted metabolomics

## Abstract

**Background/Objectives**: Although donepezil, a reversible acetylcholinesterase inhibitor, has been in use since 1996, its metabolic characteristics remain poorly characterized. Therefore, this study aims to investigate the in vivo metabolism of donepezil using liquid chromatography–high-resolution mass spectrometry (LC-HRMS) based on a molecular networking (MN) approach integrated with a non-targeted metabolomics approach. **Methods**: After the oral administration of donepezil (30 mg/kg) in rats, urine, feces, and liver samples were collected for LC-HRMS analysis. Chromatographic and spectrometric data were processed through MN and multivariate data analysis to identify the in vivo metabolites of donepezil. **Results**: A total of 50 metabolites were characterized, including 23 newly identified metabolites. Donepezil was biotransformed by *O-*demethylation, *N-*debenzylation, and hydroxylation, and these metabolites are further conjugated with glucuronic acid and sulfurous acid. *N-*Desbenzyldonepezil (**M4**), didesmethyldonepezil (**M5**), and *N-*desbenzyldonepezil (**M4**) were identified as the most abundant metabolites in urine, feces, and liver samples, respectively. **Conclusions**: The metabolic characteristics of donepezil in rats were comparable to those in humans, indicating that a rat is a reliable model for studying donepezil metabolism. This study indicates that a MN approach combined with a metabolomics approach is a reliable tool to identify unknown metabolites of drugs and drug candidates.

## 1. Introduction

Donepezil is a reversible acetylcholine inhibitor primarily prescribed for patients with Alzheimer’s disease with mild-to-moderate cognitive impairment. Donepezil enhances the efficiency of nerve signal transmission and improves the cognitive function of patients by reversibly binding to acetylcholinesterase [1,2].

Donepezil is known to be metabolized by CYP3A and CYP2D6 to form *O-*demethylation, *N-*debenzylation, hydroxylation, and *N-*oxidation metabolites [3,4]. These metabolites are further conjugated with glucuronic acid or sulfurous acid and excreted through the urine and bile [4,5,6] (Figure S1). In detail, Tiseo et al. [5] investigated donepezil metabolism using ^14^C-labeled donepezil in humans and they identified *O-*desalkyldonepezils, *N-*desbenzyldonepezil, donepezil*-N-*oxide, and *O-*desmethyldonepezil glucuronides as major metabolites. Matsui et al. report significant advances in understanding donepezil metabolism [6]. They identified 15 metabolites, including six novel ones (two *O-* desmethyl sulfates, two *O-*didesmethyldonepezil glucuronides, *O-*didesmethyl-hydroxydonepezil, *N-*desbenzyl-hydroxydonepezil, and benzene ring-hydroxylated*-O-*desmethyldonepezil along with its sulfate conjugate) in rat urine and feces.

Several post-acquisition data analysis strategies for metabolite identification have been developed. Non-targeted metabolomics combined with statistical tools, is commonly utilized in drug metabolism profiling, as seen with amiodarone [7], itraconazole [8], and sildenafil [9]. Additionally, molecular networking (MN), which determines the structural similarity between molecules based on spectral similarity, has emerged as an efficient tool for metabolite identification [10,11]. It has been applied to drugs such as anamorelin [12], sildenafil [10], and seongsanamide A [13], as well as designer drugs [14].

In our previous work, a metabolomics approach combined with MN was successfully applied to investigate the phase I metabolites of donepezil in vitro, resulting in the identification of 14 new metabolites, demonstrating the effectiveness of this approach [4]. Although in vivo metabolites of donepezil have been identified in rats and humans [3,5,6], these identifications were based solely on conventional methods that rely on interpreting mass fragmentation patterns in the mass spectra of metabolite candidates. Therefore, this study aims to investigate the metabolic pathway of donepezil in rats using non-targeted metabolomics and MN approaches.

## 2. Materials and Methods

### 2.1. Reagents

Donepezil hydrochloride was obtained from Sigma-Aldrich (St. Louis, MO, USA). 3-Hydroxydonepezil and desbenzyldonepezil were sourced from the Tokyo Chemical Industry (Tokyo, Japan) and Cayman (Ann Arbor, MI, USA), respectively. All solvents for liquid chromatography–mass spectrometry were provided by Fisher Scientific Co. (Pittsburgh, PA, USA) with the highest grade commercially available.

### 2.2. Animal Experiment

Animal studies were conducted in accordance with the guidelines of the Kyungpook National University Animal Care and Use Committee. Male Sprague Dawley (SD) rats (6–8 weeks old, weighing 200–250 g) were obtained from SAMTAKO (Osan, Republic of Korea). The rats were fasted overnight before dosing. Animals were administered donepezil orally at 30 mg/kg, based on the LD_50_ value of donepezil in rats [15], while the controls were administered an equivalent volume of distilled water (*n* = 6). All animals were housed individually in metabolic cages equipped with urine and feces separators. Feeding was resumed 4 h after donepezil administration. Urine and feces samples were collected over a 24 h period. All rats were sacrificed, and their livers were harvested for analysis. Samples were stored separately at −80 °C until analysis.

### 2.3. Sample Preparations

Feces and liver samples were homogenized with water at 5× and 1.5× the total volume of each sample, respectively. To select the optimum extraction solvent for the maximum extraction of donepezil and its metabolites from biological samples, three solvents were evaluated: (1) methanol (MeOH), (2) acetone:MeOH (3:7, *v/v*), and (3) acetonitrile (ACN):MeOH (1:1, *v/v*), as reported in previous studies [6,16,17,18]. Urine (200 µL), fecal, and liver homogenates (400 µL) were mixed with cold extraction solvents at twice their volume. The mixtures were vortexed for 10 s, and then centrifuged at 12,700× *g* (4 °C, 10 min). The aliquots were dried to completion, reconstituted with 100 μL of 50% aqueous MeOH, vortexed, and filtered. The precipitates of the liver sample were used for protein quantification.

### 2.4. LC-MS/MS Analysis

Chromatographic and spectral data were acquired using a DIONEX UltiMate 3000 ultra-high-performance liquid chromatography system coupled with a Q Exactive Focus hybrid quadrupole-Orbitrap mass spectrometer (Thermo Fisher Scientific Inc., Waltham, MA, USA). Electrospray ionization in positive mode was used for mass spectrometry. The Kinetex XB-C18 column (Phenomenex Corporation, Torrance, CA, USA, 100 × 2.1 mm, 2.6 µm, 100 Å) was employed to separate donepezil and its metabolites. The mobile phases consisted of water with 0.1% formic acid (solvent A) and acetonitrile with 0.1% formic acid (solvent B), delivered at a flow rate of 0.2 mL/min. The composition of the mobile phase was modified as follows: 0–12 min (B: 10–35%), 12–15 min (B: 35–90%), 15–15.1 min (B: 90–10%), and 15.1–18 min (B: 10%) [4]. Full scan and data-dependent MS/MS (ddMS^2^) modes were used to obtain the mass spectral data. The conditions were based on a previously published method with minor modifications [4]. In addition, the following conditions were used for parallel reaction monitoring (PRM), targeting MS^2^ of precursors in the inclusion list of expected metabolites: resolution, 17,500; collision energy, 30 eV; AGC target, 5 × 10^4^; and maximum injection time, 100 ms. The inclusion list included the precursor ion mass of predicted metabolites. Chromatograms and mass spectra were obtained, before being analyzed using the Thermo Xcalibur 4.1 software (Thermo Fisher Scientific Inc.).

### 2.5. Multivariate Analysis

After converting the data file (.raw) to an .mzML file, it was processed using MZmine open-source software (version 2.53) for peak detection, chromatogram deconvolution, isotope grouping, and feature alignment. The processed CSV files, including feature tables (*m/z*, retention time, and peak area values), were used for multivariate analysis. Three normalization methods were evaluated for each sample as follows: no normalization, volume, and creatinine peak area (*m/z* 114.0662) for urine; no normalization, wet weight, and total MS signal for feces; and no normalization, protein amount, and wet weight for liver [19]. Multivariate analysis was performed using SIMCA software (version 14.1, Umetrics AB, Umea, Sweden). Principal component analysis and orthogonal partial least squares discriminant analysis (OPLS-DA) were conducted on Pareto-scaled data to identify significant differences between groups. The S-plot was used to assess the relative importance of variables in group separation. Variables with *p (corr)* > 0.4 and VIP (variable importance in the projection) > 1 in the S-plot were considered candidate metabolites of donepezil [20]. The structure of the metabolite candidates was identified by comparing the retention time and MS/MS fragmentation pattern with those of standard reagents of donepezil and its metabolites.

### 2.6. Molecular Networking

The MN was developed using the GNPS (Global Natural Products Social Molecular Networking) website (http://gnps.ucsd.edu) [4,13]. The MZmine data were evaluated using the feature networking workflow supplied by GNPS. The following parameters were set to establish the donepezil network: precursor ion mass tolerance of 0.02 Da and fragment ion mass tolerance of 0.02 Da. Nodes were connected when the cosine score exceeded 0.5 and the MS/MS spectrum contained at least six matched peaks. While a cosine score cutoff of 0.7 is commonly recommended, a 0.5 threshold was specifically used here to enhance the discovery of more metabolites [21].

## 3. Results and Discussion

To determine the optimum solvents for extracting donepezil and its metabolites from biological samples, three solvents (methanol, acetone:methanol (3:7, *v/v*), and acetonitrile:methanol (1:1, *v/v*)), which had been previously used in similar analyses [6,16,18], were tested in triplicates. The extraction efficiency was assessed by comparing the peak intensities of donepezil and its four representative metabolites (hydroxydonepezil, *O-*desmethyldonepezil, *O-*desmethyldonepezil glucuronide, and *O-*desmethyldonepezil sulfate) in urine, feces, and liver tissue extracts. Acetonitrile:methanol (1:1, *v/v*) showed the highest peak intensities in urine and feces extracts, whereas acetone:methanol (3:7, *v/v*) showed the highest peak intensities in liver extracts (Figure S2).

### 3.1. Profiling of Donepezil Metabolites Using Metabolomic and Molecular Networking Approaches

**Figure 1:**
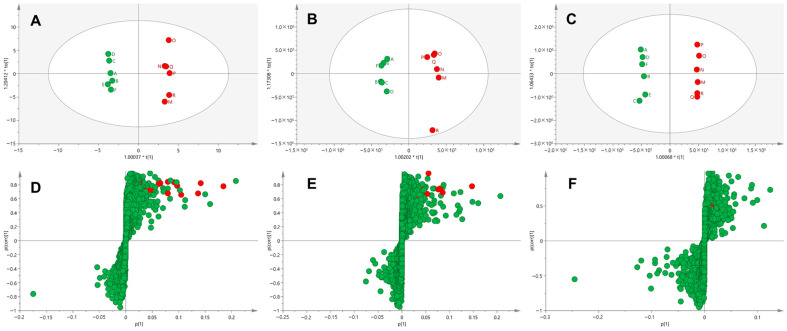
Multivariate analysis of donepezil metabolites in urine (**A**,**D**), feces (**B**,**E**), and liver (**C**,**F**). Score plots were generated using OPLS−DA based on the LC-HRMS data of samples. Loading S−plots were generated using OPLS−DA based on the LC-HRMS data of urine (**D**), feces (**C**), and liver (**F**) samples. The *p* [1] values represent the relative abundance of ions, while *p* (corr) [1] values indicate the interclass difference. Green dots on the S−plot represent variables (mass spectral data) that reflect the influence of each variable in the two groups (control vs. donepezil). Variables farthest from the origin in the S−plot are identified as potential donepezil metabolites (red dots). Details of data processing and model construction are provided in Section 2. Abbreviations: OPLS−DA, orthogonal partial least square−discriminant analysis; LC-HRMS, liquid chromatography–high-resolution mass spectrometry.

**Figure 2:**
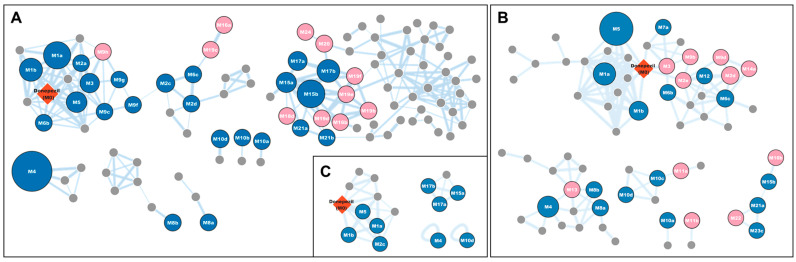
Representative non-targeted molecular network of the MS/MS spectra obtained by the LC-HRMS analysis of the urine (**A**), feces (**B**), and liver (**C**). The node size is proportional to MS^1^ ion intensities, while the thickness between nodes is proportional to the cosine score. Unlabeled gray circles are variables that are networked with donepezil but were confirmed not to be donepezil metabolites based on MS/MS spectral analysis. (Known metabolites ⬤ and newly identified metabolites ⬤). The FBMN jobs for urine (**A**), feces (**B**), and liver (**C**) data are available on the GNPS website at the following links: https://gnps.ucsd.edu/ProteoSAFe/status.jsp?task=1ad16a70f54b47499aff9f211d741d87 accessed on 13 November 2024 for urine, https://gnps.ucsd.edu/ProteoSAFe/status.jsp?task=9e79043db66e4550bac348a66317e306 accessed on 13 November 2024 for feces, and https://gnps.ucsd.edu/ProteoSAFe/status.jsp?task=f6f04bcfa37645b699640901f9fc230e accessed on 13 November 2024 for liver. Abbreviation: LC-HRMS, liquid chromatography–high-resolution mass spectrometry.

The in vivo metabolism of donepezil in treated rats was investigated by identifying its phase I and II metabolites in urine, feces, and liver tissues. A metabolomic and MN approach was used for comprehensive in vivo metabolite identification. Chromatographic raw data were normalized using the creatinine peak area (*m/z* 114.0662) for urine samples and weight values for feces and liver tissues [19,22,23,24]. The control and sample groups were clearly separated in the OPLS-DA score plot (Figure 1A–C), and the S-plot was used to identify the variables driving this separation (Figure 1D–F). The cut-off values were set to *p (corr)* > 0.4 and VIP > 1.0, reducing the number of donepezil metabolite candidates to 1133 in urine, 1228 in feces, and 502 in liver tissues. Additionally, a molecular network was created using the GNPS platform for the high-resolution mass spectrometry raw data processed via MZmine (Figure 2) [10]. Each node, representing an ion with retention time, was labeled with the identified metabolite name and clustered based on MS/MS spectral similarities [13]. Two major clusters linked to donepezil (M0) or its glucuronide conjugates were identified, along with *N-*debenzylation-related clusters containing *N-*desbenzyl-donepezil (**M4**) (Figure 2).

**Figure 3:**
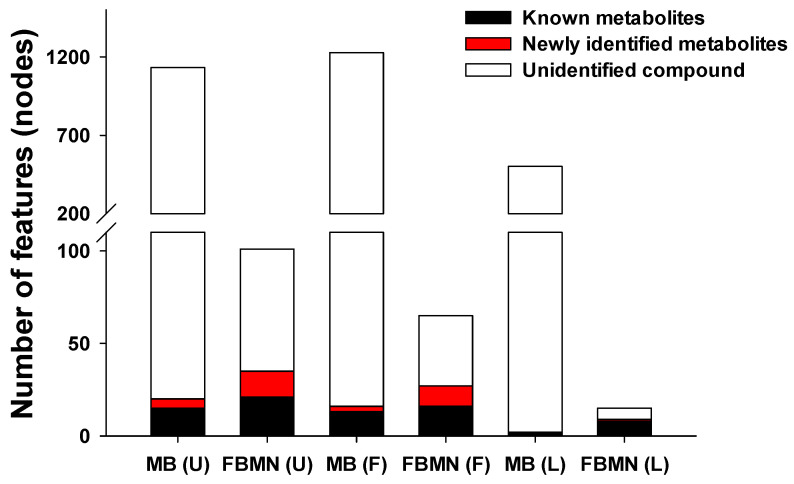
Summary of the MS features detected by non-targeted metabolomics and FBMN for each sample type (U, urine sample; F, feces sample; L, liver sample). Abbreviations: MB, metabolomics; FBMN, feature-based molecular networking.

The metabolomic approach identified numerous metabolite candidates (1133, 1228, and 502 candidates for urine, feces, and liver tissues, respectively). Among them, only 20, 16, and 2 were identified as potential donepezil metabolites in urine, feces, and liver tissues, respectively (Figure 3). However, the MN approach, which analyzes MS/MS spectral similarities, identified significantly fewer metabolite candidates compared than the metabolomic approach (101, 65, and 15 candidates for urine, feces, and liver tissues, respectively), allowing for a more efficient identification of metabolite structures [4,10,13]. Finally, 35, 27, and 9 potential donepezil metabolites were identified in urine, feces, and liver tissues, respectively, using the MN approach (Figure 3).

### 3.2. Phase I Metabolites Structure Identification

Metabolite structures were identified using accurate mass, product ion scan mass (MS/MS) spectra, and fragment ion patterns. Metabolites **M1a**, **M1b**, **M2c**, **M3**, and **M4** were confirmed by comparing their retention time and MS/MS spectra with that of a standard compound. Table 1 summarizes the retention time, theoretical and measured masses, mass errors (within 3.0 ppm), chemical formula, and reaction types of donepezil and its metabolites. Figures S3 and S4 depict the representative extracted ion chromatograms and MS/MS spectra of metabolites.

To identify metabolite structures, the structural properties of the parent drug were determined. Donepezil (**M0**, 10.2 min) showed a protonated molecular ion at *m/z* 380.2213 (mass error < 1.9 ppm). Collision-induced fragmentation at *m/z* 380.2213 produced a base peak at *m/z* 91.0545, corresponding to a dissociated benzylic carbocation (Figure S4A). The characteristic fragment ions at *m/z* 362.2103, 288.1587, and 151.0750, previously reported [4], were also detected. The metabolites **M1a** (8.46 min), **M1b** (8.72 min), **M2c** (8.21 min), **M3** (11.02 min), and **M4** (5.59 min) were identified as 6*-O-*desmethyldonepezil, 5*-O-*desmethyldonepezil, 3-hydroxydonepezil, donepezil*-N-*oxide, and *N-*desbenzyldonepezil, respectively, using co-chromatography and MS/MS spectra of authentic standards (Figure S4B,C,F,I,J).

Five hydroxylation metabolites (**M2a**–**M2e**, *m/z* 396.2161) were identified in urine, feces, and liver extracts. Among them, the MS/MS spectra of **M2a**, **M2c**, and **M2d** (Figure S4D,F,G) matched those of piperidine ring-, cyclopentanone ring-, and benzyl ring-hydroxylated donepezil, respectively, as previously reported [4]. **M2b** produced fragment ions at *m/z* 91.0544 and 151.0749, indicating no metabolic changes in the benzyl and dimethoxyindanone groups (Figure S4E). The characteristic fragment ion of **M2b** observed at *m/z* 204.1377 represents a 16-Da increase in the 1-benzyl-4-methylpiperidine group. Similarly to **M2d**, the metabolite **M2e** also produced fragment ions at *m/z* 151.0751 and 107.0492, indicating no metabolic changes in the dimethoxyindanone group and benzyl ring hydroxylation, respectively (Figure S4H).

The MS/MS spectra of **M5** (Figure S4K) were similar to those of *O-*didesmethyldonepezil reported in a previous study [4]. Five hydroxylated *O-*desmethyl metabolites (**M6a**, **M6b**, **M6c**, **M7a**, and **M7b**, *m/z* 382.2013) were detected in the extracted ion chromatograms of urine extracts, with their MS/MS spectra matching those of 6*-O-*desmethyl-piperidine ring-hydroxylated donepezil, 5*-O-*desmethyl-piperidine ring-hydroxylated donepezil, *O-*desmethyl-benzene ring-hydroxylated donepezil, 6*-O-*desmethyldonepezil *N-*oxide, and 5*-O-*desmethyldonepezil *N-*oxide, respectively (Figure S4L–P) [4]. The MS/MS spectra of **M8a** and **M8b** (Figure S4Q,R) were similar to those of 6*-O-*desmethyl*-N-*desbenzyldonepezil and 5*-O-*desmethyl*-N-*desbenzyldonepezil, respectively, as reported previously [4].

Nine dihydroxylation metabolites (**M9a**–**M9i**, *m/z* 412.2118) were detected in the extracted ion chromatograms of urine and/or feces extracts (Figure S3). **M9a**, **M9b**, **M9c**, and **M9f** produced fragment ions at *m/z* 91.0542, indicating no metabolic changes in the benzyl groups (Figure S4S,T,U,X). The absence of peaks at *m/z* 167.0703 indicates that hydroxylation has not occurred on the benzene portion of the dimethoxyindanone group. **M9a** and **M9b** produced characteristic fragment ions at *m/z* 204.1383, which is a 32-Da increase over the *N-*benzylpiperidine group (*m/z* 172.1121) found in **M9c** and **M9f** (Figure S4U,X). **M9d** produced characteristic fragment ions at *m/z* 123.0439, indicating a 32-Da increase over the benzyl group (Figure S4V). In addition, the fragment ion at *m/z* 151.0753 and 290.1742 confirmed no hydroxylation in the dimethoxyindanone group. **M9e** produced characteristic fragment ions at *m/z* 167.0702 and 107.0492, indicating that hydroxylation occurs on the benzene part of the dimethoxyindanone and benzyl groups, respectively (Figure S4W). **M9g** produced fragment ions at *m/z* 91.0544 and 151.0754, indicating no metabolic changes in the benzyl and dimethoxyindanone groups (Figure S4Y). Generally, *N-*oxide metabolites have longer retention times than that of the parent drug [4,25]. In this study, donepezil *N-*oxide (**M3**) and *O-*desmethyl*-N-*oxide (**M7**) also had longer retention times than those of donepezil and *O-*desmethyldonepezil (**M1**), respectively. The retention times of **M9h** and **M9i** (9.09 and 9.53 min) exceeded those of hydroxydonepezil (**M2**, 7.73~8.81 min), indicating that **M9h** and **M9i** have an *N-*oxide structure. **M9h** and **M9i** produced fragment ions at *m/z* 91.0544, indicating no metabolic changes in the benzyl groups (Figure S4Z,AA). Overall, **M9h** and **M9i** are identified as hydroxydonepezil*-N-*oxide, with their predicted fragment ion structures (Figure S5).

**Figure 4:**
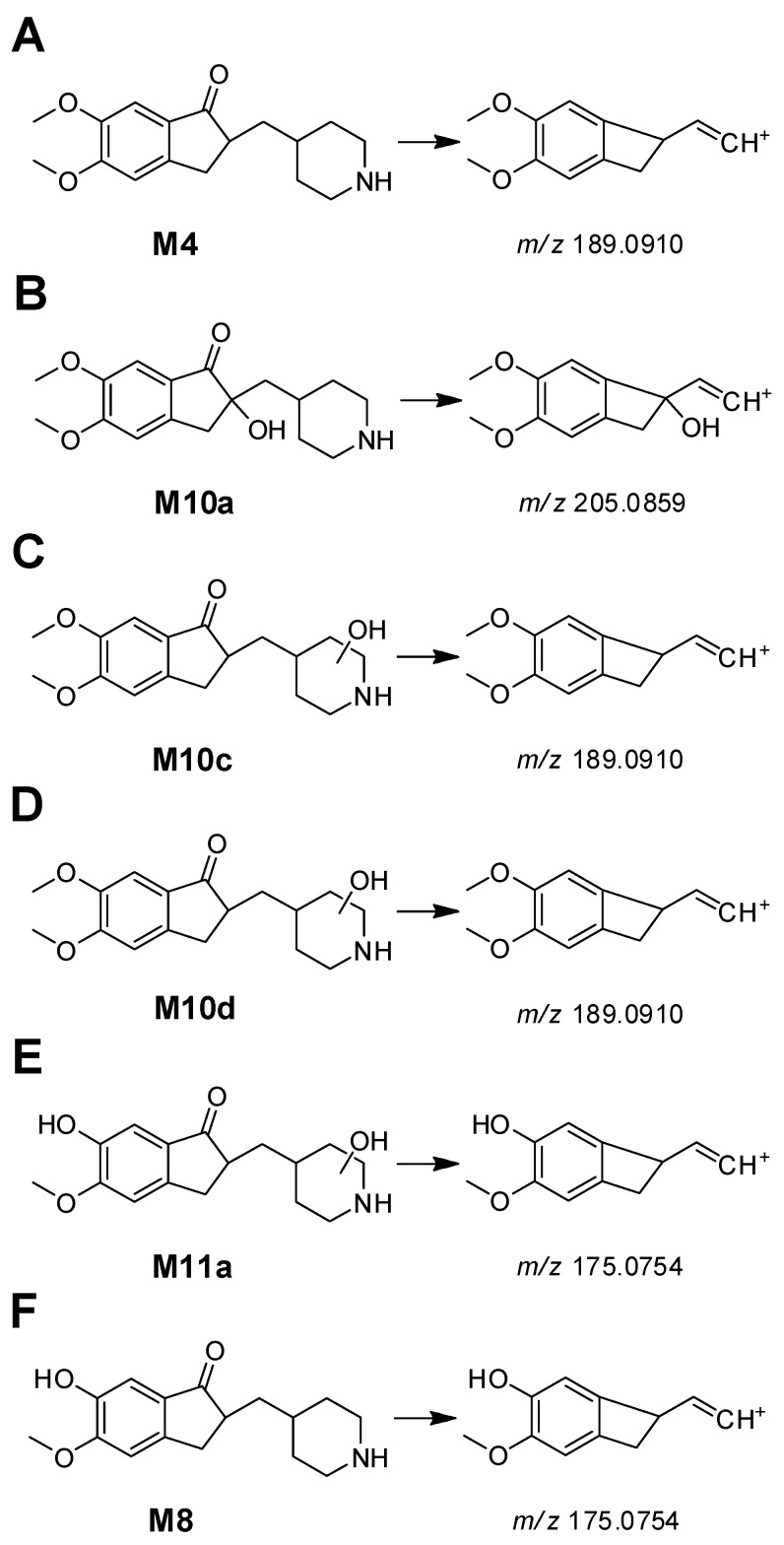
Fragmentation schemes of *N-*desbenzyldonepezil (**A**), 2-hydroxyl*-N-*desbenzyldonepezil (**B**), piperidine ring-hydroxylated*-N-*desbenzyldonepezil (**C**,**D**), *O-*desmethyl*-N-*desbenzylhydroxydonepezil (**E**), and *O-*desmethyl*-N-*desbenzyldonepezil (**F**).

Four **M10** metabolites (**M10a**~**M10d**) had a protonated molecular ion at *m/z* 306.1700, matching the mass of the hydroxylation *N-*desbenzyldonepezil (**M4**) metabolite. **M10a**, **M10c**, and **M10d** produced fragment ions at *m/z* 151.0754, indicating no metabolic changes in the dimethoxyindanone group (Figure S4AB,AD,AE). **M10a** produced fragment ions at *m/z* 205.0853, while **M4**, **M10c**, and **M10d** produced fragment ions at *m/z* 189.0910 (loss of oxygen from *m/z* 205.0859). **M10a** also produced fragment ions at *m/z* 84.0811 and 96.0809, indicating no metabolic changes in the methylpiperidine group (Figure S4AB), while **M10b** and **M10c** produced fragment ions at *m/z* 114.0913, indicating hydroxylation of the methylpiperidine group and piperidine ring, respectively (Figure S4AC,AD). The structures of fragment ions at m/z 205.0859 and 189.0910 are presented in Figure 4. Based on these results, **M10a** was identified as 2-hydroxy*-N-*desbenzyldonepezil, **M10b** as methylpiperidine ring-hydroxylated*-N-*desbenzyldonepezil, and **M10c** and **M10d** as piperidine ring-hydroxylated*-N-*desbenzyldonepezil.

**M11a** and **M11b** had a protonated molecular ion at *m/z* 292.1543, identical to the mass of the hydroxylated *O-*desmethyl*-N-*desbenzyldonepezil (**M8**) metabolite. **M11a** produced fragment ions at *m/z* 137.0594, indicating no metabolic changes in the indanone group (Figure S4AF), and produced fragment ions at *m/z* 175.0745 (corresponding to the loss of methylene from *m/z* 189.0910 found in **M10c** and **M10d**) (Figure 4C,D). These fragment ions were also present in the MS/MS spectrum of *O-*desmethyl*-N-*desbenzyldonepezil (**M8**) (Figure S4Q,R and Figure 4F).

**M12** and **M13** had protonated molecular ions at *m/z* 368.1856 and 262.1438, identical to the masses of the hydroxylated and *N-*debenzylated metabolites of *O-*didesmethyldonepezil (**M5**), respectively. **M12** produced characteristic fragment ions at *m/z* 107.0492 and 262.1430, indicating benzene ring hydroxylation and no changes in the dihydroxyindanone and methylpiperidine groups, respectively (Figure S4AH). **M13** produced fragment ions at *m/z* 82.0654 and 177.0541, confirming the existence of the piperidine and 4-methyl-dihydroxyindanone groups, respectively (Figure S4AI). Consequently, **M12** and **M13** were identified as *O-*didesmethylhydroxydonepezil and *O-*didesmethyl*-N-*desbenzyldonepezil, respectively. The three **M1**4 metabolites had protonated molecular ions at *m/z* 398.1962 and underwent *O-*demethylation and dihydroxylation (Figure S4AJ,AK,AL). **M14a**, **M14b**, and **M14c** showed characteristic fragment ions at *m/z* 107.0492, 123.0439, and 91.0545, respectively, indicating hydroxylation, dihydroxylation, and no modification of the benzene ring. **M14a** and **M14c** showed additional characteristic fragment ions at *m/z* 137.0594 and 172.1121, indicating no metabolic changes in the indanone and benzylpiperidine groups, respectively (Figure S4AJ,AL).

### 3.3. Phase II Metabolites Structure Identification

In general, collision-induced fragmentation of glucuronide and sulfate conjugates results in product ions caused by the typical neutral losses of glucuronide (176 Da) and sulfate (80 Da), respectively [26,27,28].

**M15** and **M16** showed protonated molecular ions at *m/z* 542.2385 and 572.2490, corresponding to the masses of metabolites formed glucuronidation of *O-*desmethyldonepezil (**M1**) and hydroxydonepezil (**M2**), respectively. They were identified as *O-*desmethyldonepezil glucuronide and hydroxydonepezil glucuronide by the loss of the glucuronosyl moiety (176 Da), forming the aglycone ion at *m/z* 366.2054 (Figure S4AM,AN) and *m/z* 396.2169 (Figure S4AO–AQ), respectively. **M15a** (5.85 min) and **M15b** (6.10 min) were identified as 6*-O-*desmethyldonepezil glucuronide and 5*-O-*desmethyldonepezil glucuronide, respectively, based on differences in retention time (Figure S3). **M16a** was identified as benzene ring-hydroxydonepezil glucuronide based on a characteristic fragment ion at *m/z* 107.0492 (hydroxytoluene). The exact glucuronide site in **M16b** and **M16c** could not be determined due to limited fragment ion formation.

**M17** and **M18** had protonated molecular ions at *m/z* 528.2228 and 544.2177, identical to the masses of metabolites that underwent glucuronidation in *O-*didesmethyldonepezil (**M5**) and *O-*didesmethyl-hydroxydonepezil (**M12**), respectively. They were identified as *O-*didesmethyldonepezil glucuronide and *O-*didesmethyl-hydroxydonepezil glucuronide by the loss of the glucuronosyl moiety (176 Da), forming aglycone ions at *m/z* 352.1907 (Figure S4AR,AS) and *m/z* 368.18556 (Figure S4AT–AW), respectively. **M18a** and **M18b** were identified as benzene ring-hydroxy*-O-*didesmethyldonepezil glucuronide based on two characteristic fragment ions at *m/z* 107.0491 (hydroxytoluene) and 438.1746 (1-(4-methylpiperidyl)-5,6-dihydroxyindanone), whereas **M18c** and **M18d** were identified as indanone ring or methylpiperidine hydroxylated *O-*didesmethyldonepezil glucuronide based on a characteristic fragment ion at *m/z* 91.0543 (toluene).

Seven **M19** (**M19a**~**M19g**) and **M20** had protonated molecular ions at *m/z* 558.2334 and 452.1915, identical to the masses of metabolites that underwent glucuronidation in *O-*desmethylhydroxydonepezil (**M6**) and *O-*desmethyl*-N-*desbenzyldonepezil (**M8**), respectively. They were identified as *O-*desmethylhydroxydonepezil glucuronide and *O-*desmethyl*-N-*desbenzyldonepezil glucuronide by the loss of the glucuronosyl moiety (176 Da), forming aglycone ions at *m/z* 382.2013 (Figure S4AX–BD) and 276.1587 (Figure S4BE), respectively. **M19a** and **M19c** were identified as benzene ring-hydroxy*-O-*desmethyldonepezil glucuronide based on a characteristic fragment ion at *m/z* 107.0491 (hydroxytoluene), whereas **M19b** and **M19d** were identified as indanone ring or methylpiperidine hydroxylated *O-*desmethyldonepezil glucuronide based on a characteristic fragment ion at *m/z* 91.0542 (toluene). The retention times of **M19e**, **M19f**, and **M19g** (6.10, 6.53, and 6.81 min, respectively) were longer than those of *O-*desmethyldonepezil glucuronide (**M15**, 5.85, and 6.10 min, respectively), indicating that **M19e**, **M19f**, and **M19g** have *N-*oxide structures and produced fragment ions at *m/z* 91.0542, indicating no metabolic changes in the benzyl groups (Figure S4BB–BD). Based on these results, **M19e**, **M19f**, and **M19g** were identified as *O-*desmethyldonepezil*-N-*oxide glucuronide. The exact attachment site of glucuronide could not be determined because of limited fragment ion formation.

**Figure 5:**
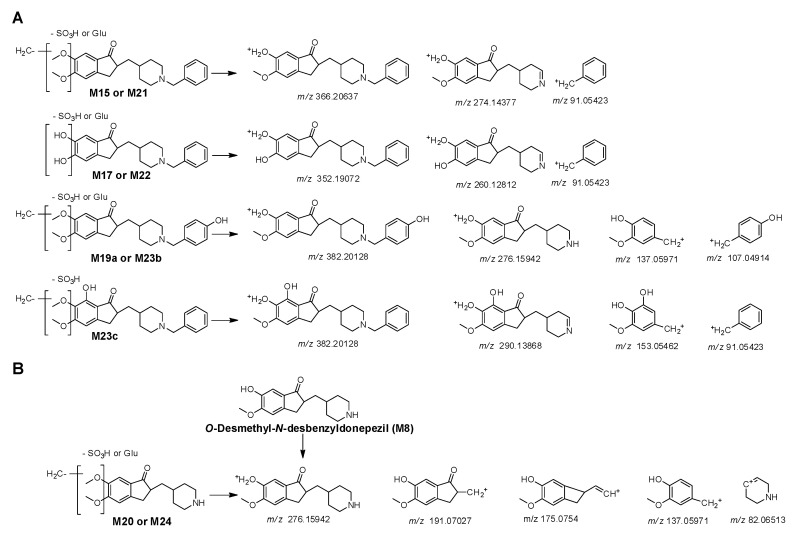
Fragmentation schemes of donepezil conjugates ((**A**), *O-*desmethyldonepezil glucuronide (**M15**), *O-*desmethyldonepezil sulfate (**M21**), *O-*didesmethyldonepezil glucuronide (**M17**), *O-*didesmethyldonepezil sulfate (**M22**), *O-*desmethylhydroxydonepezil glucuronide (**M19**), and *O-*desmethylhydroxydonepezil sulfate (**M23**)) and *N-*desbenzyldonepezil conjugates ((**B**), *O-*desmethyl*-N-*desbenzyldonepezil sulfate (**M24**) and *O-*desmethyl*-N-*desbenzyldonepezil glucuronide (**M20**)).

**M21** and **M22** had protonated molecular ions at *m/z* 446.1632 and 432.1475, identical to the mass of metabolites that underwent sulfate conjugation in *O-*desmethyldonepezil (**M1**) and *O-*didesmethyldonepezil (**M5**), respectively. They were identified as *O-*desmethyldonepezil sulfate and *O-*didesmethyldonepezil sulfate by the neutral loss of 80 Da, forming the fragment ion at *m/z* 366.2054 (Figure S4BF,BG) and 352.1898 (Figure S4BH), respectively. **M23** and **M24** had protonated molecular ions at *m/z* 462.1581 and 356.1162, matching the masses of metabolites that underwent sulfate conjugation in *O-*desmethylhydroxydonepezil (**M6**) and *O-*desmethyl*-N-*desbenzyldonepezil (**M8**), respectively. They were identified as *O-*desmethylhydroxydonepezil sulfate and *O-*desmethyl*-N-*desbenzyldonepezil sulfate by the neutral loss of 80 Da, forming the fragment ion at *m/z* 382.2013 (Figure S4BJ,BK) and 276.1586 (Figure S4BL), respectively. **M23a** and **M23b** were identified as benzene ring-hydroxy*-O-*desmethyldonepezil sulfate based on a characteristic fragment ion at *m/z* 107.0492 (hydroxytoluene) (Figure S4BI,BJ), whereas **M23**c was identified as indanone ring-hydroxylated *O-*desmethyldonepezil sulfate based on two characteristic fragment ions at *m/z* 91.0544 (toluene) and 153.0542 (Figure S4BK). The fragmentation patterns of sulfate conjugates were similar to those of their corresponding glucuronide conjugates (Figure 5A). **M24** produced fragment ions at *m/z* 137.0593, 175.0750, and 191.0697 (Figure S4BL), which were also present in the MS/MS spectrum of *O-*desmethyl*-N-*desbenzyldonepezil (**M8**) and *O-*desmethyl*-N-*desbenzyldonepezil glucuronide (**M20**) (Figure 5B).

### 3.4. Distribution of Donepezil Metabolites in Urine, Feces, and Liver Samples

**Figure 6:**
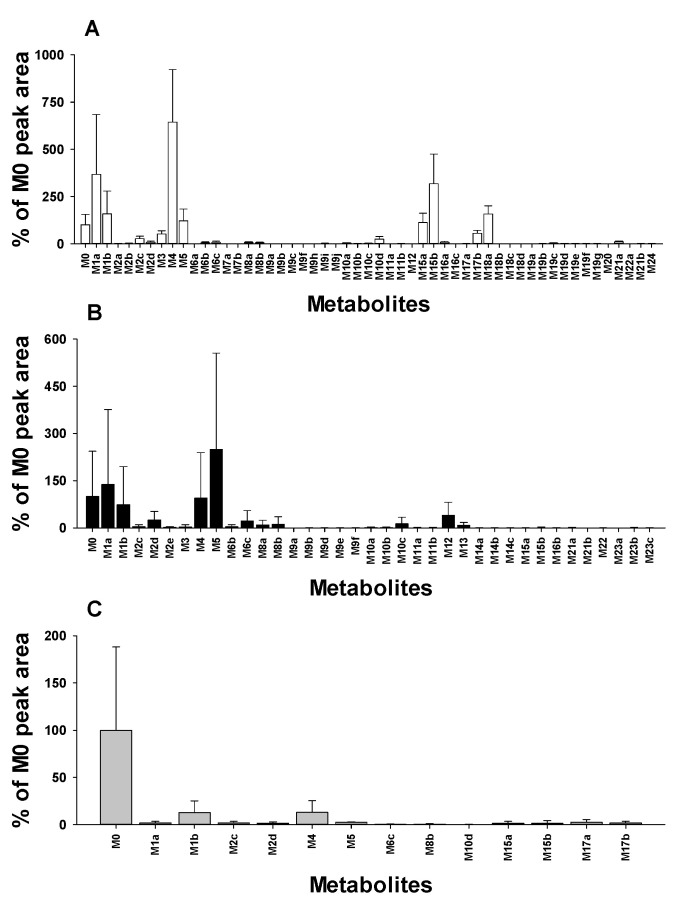
Relative distribution of donepezil metabolites showing the abundance of the metabolites present in rat urine (**A**), feces (**B**), and liver (**C**) compared to donepezil (**M0**). The relative abundance of donepezil metabolites was determined by averaging results from six replicated experiments.

A total of 50 metabolites, including 23 newly identified ones, were identified through MN- and metabolomics-guided profiling in rat samples (Table 1). *N-*desbenzyldonepezil (**M4**) and didesmethyldonepezil (**M5**) were identified as the most abundant metabolites in urine and feces, respectively, while *N-*desbenzyldonepezil (**M4**) was also predominant in liver samples (Figure 6). Furthermore, these metabolites were the predominant metabolites in urine, feces, and liver tissues after a single oral administration of donepezil in rats [6]. *N-*desbenzyldonepezil (**M4**), 6*-O-*desmethyldonepezil (**M1a**), 5*-O-*desmethyldonepezil glucuronide (**M15b**), didesmethyl-hydroxydonepezil glucuronide (**M18a**), didesmethyldonepezil (**M5**), 5*-O-*desmethyldonepezil (**M1b**), 6*-O-*desmethyldonepezil glucuronide (**M15a**), didesmethyldonepezil glucuronide (**M17b**), and donepezil*-N-*oxide (**M3**) were the major metabolites in urine samples (Figure 6A). Among them, **M4**, **M1a**, **M1b**, **M5**, and **M3** were predominant in urine after a single oral administration of donepezil in rats [6]. In addition, **M4**, **M1a**, **M1b**, and **M3** were the predominant metabolites in plasma following the oral administration of donepezil to Sprague Dawley rats [18]. In feces, didesmethyldonepezil (**M5**), 6*-O-*desmethyldonepezil (**M1a**), *N-*desbenzyldonepezil (**M4**), 5*-O-*desmethyldonepezil (**M1b**), didesmethyl-hydroxydonepezil (**M12**), *O-*desmethylhydroxydonepezil (**M6c**), and benzyl-hydroxylated donepezil (**M2d**) were the major metabolites of donepezil (Figure 6B). These metabolites were also predominant in feces after a single oral administration of donepezil in rats [6]. **M1**, **M3**, **M4**, and **M15** were also predominant metabolites in urine and feces after a single oral administration of donepezil in healthy volunteers [5]. *N-*Desbenzyldonepezil (**M4**) and 5*-O-*desmethyldonepezil (**M1b**) were the major metabolites in liver tissues (Figure 6C).

Overall, the metabolic characteristics of donepezil in rats were comparable to those in humans [5,18]. All metabolites of donepezil in humans and human liver microsomes were identified in rats (Table 1 and Figure S1) [4,5]. From a drug development perspective, the primary concern is the presence of human-specific metabolites not found in animals [29]. This study shows that donepezil metabolism is similar in animals and humans; therefore, metabolic data from rat models is relevant to humans.

## 4. Conclusions

**Figure 7:**
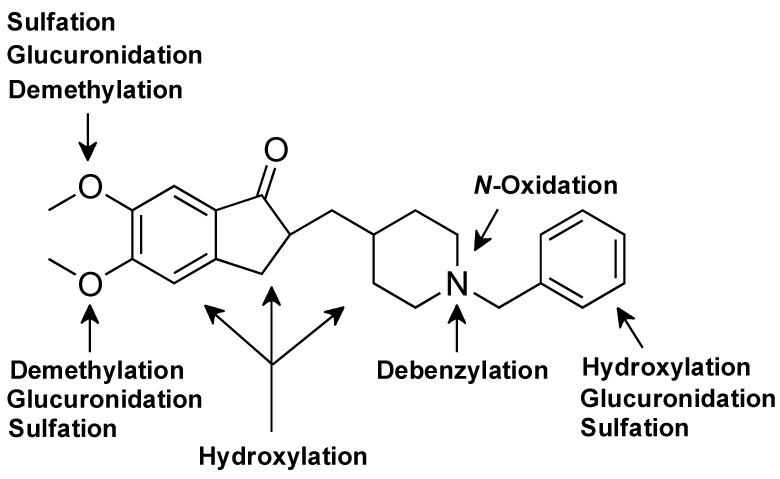
Chemical structure of donepezil showing the locations of metabolic biotransformations in rats.

**Figure 8:**
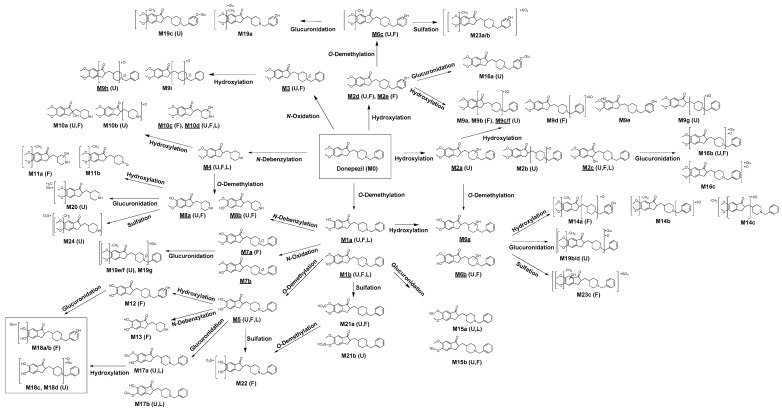
Proposed metabolic pathways of donepezil in rats (urine: U; feces: F; and liver: L). Metabolites that were also identified in vitro studies are underlined. *O*-desmethyl-3-hydroxydonepezils, which had been identified in vitro studies, were not identified through MN- and metabolomics-guided profiling in rat samples.

In this study, the in vivo metabolism of donepezil in rats was investigated using an MN combined with non-targeted metabolomics and high-resolution mass spectrometry. The structures of the metabolites were identified based on precise mass and fragmentation patterns. A total of 50 metabolites (36 metabolites in urine, 28 metabolites in feces, and 9 metabolites in liver) were identified, among which 23 metabolites (**M2b**, **M9d**, **M9g**, **M10a~b**, **M11a~b**, **M13**, **M14a**, **M16a~b**, **M18a~b**, **M18d**, **M19b~f**, **M20**, **M22**, **M23**c, and **M24**) were newly identified (Table 1). The structures of novel metabolites are primarily characterized by the addition of hydroxyl groups to positions other than the previously identified site or further conjugation reactions. The positions of these metabolic biotransformations are illustrated in Figure 7. After oral administration of donepezil in rats, it was biotransformed through five major metabolic pathways: (1) *O-*demethylation, (2) *N-*debenzylation, (3) hydroxylation, (4) glucuronidation, and (5) sulfation (Figure 8).

In conclusion, our findings indicate that both an MN and non-targeted metabolomics approach are reliable tools for identifying unknown drug metabolites. The in vivo metabolite profiling of donepezil revealed 23 novel metabolites and different metabolite distribution patterns in rat urine, feces, and liver tissues.

## Figures and Tables

**Table 1:** Summary of donepezil metabolites identified in urine, feces, and liver tissues after oral administration of donepezil to rats.

Metabolites	t_R_ (min)	[M+H]^+^		Error (ppm)	Formula (Neutral)	Reaction Type	Urine		Feces		Liver	
		Measured	Theoretical				MB ^a^	FBMN	MB	FBMN	MB	FBMN
Donepezil	10.22	380.2213	380.2220	−1.84	C_24_H_29_NO_3_	-						
**M1a**	8.46	366.2057	366.2064	−1.91	C_23_H_27_NO_3_	6-*O*-demethylation	+	+	+	+	-	+
**M1b**	8.72	366.2057	366.2064	−1.91	C_23_H_27_NO_3_	5-*O*-demethylation	+	+	+	+	+	+
**M2a**	7.73	396.2159	396.2169	−2.52	C_24_H_29_NO_4_	hydroxylation	-	+	-	-	-	-
**M2b**	8.01	396.2162	396.2169	−1.77	C_24_H_29_NO_4_	hydroxylation	+	-	-	-	-	-
**M2c**	8.21	396.2163	396.2169	−1.51	C_24_H_29_NO_4_	hydroxylation	+	+	+	+	-	+
**M2d**	8.40	396.2159	396.2169	−2.52	C_24_H_29_NO_4_	hydroxylation	-	+	+	-	-	-
**M2e**	8.81	396.2161	396.2169	−2.02	C_24_H_29_NO_4_	hydroxylation	-	-	+	+	-	-
**M3**	11.02	396.2163	396.2169	−1.51	C_24_H_29_NO_4_	*N*-oxidation	+	+	-	+	-	-
**M4**	5.59	290.1743	290.1751	−2.76	C_17_H_23_NO_3_	*N*-debenzylation	+	+	+	+	-	+
**M5**	7.12	352.1901	352.1907	−1.70	C_22_H_25_NO_3_	didemethylation	+	+	+	+	-	+
**M6a**	5.97	382.2006	382.2013	−1.83	C_23_H_27_NO_4_	6-*O*-demethylation + hydroxylation	-	-	-	-	-	-
**M6b**	6.56	382.2003	382.2013	−2.62	C_23_H_27_NO_4_	5-*O*-demethylation + hydroxylation	+	+	+	+	-	-
**M6c**	6.85	382.2003	382.2013	−2.62	C_23_H_27_NO_4_	*O*-demethylation + hydroxylation	+	+	+	+	-	-
**M7a**	9.28	382.2005	382.2013	−2.09	C_23_H_27_NO_4_	6-*O*-demethylation + *N*-oxidation	-	-	-	+	-	-
**M7b**	9.57	382.2008	382.2013	−1.31	C_23_H_27_NO_4_	5-*O*-demethylation + *N*-oxidation	-	-	-	-	-	-
**M8a**	3.75	276.1588	276.1594	−2.17	C_16_H_21_NO_3_	6-*O*-demethylation + *N*-debenzylation	+	+	+	+	-	-
**M8b**	4.02	276.1588	276.1594	−2.17	C_16_H_21_NO_3_	5-*O*-demethylation + *N*-debenzylation	+	+	+	+	-	-
**M9a**	5.79	412.2107	412.2118	−2.67	C_24_H_29_NO_5_	dihydroxylation	-	-	-	-	-	-
**M9b**	6.48	412.2108	412.2118	−2.43	C_24_H_29_NO_5_	dihydroxylation	-	**-**	-	+	-	-
**M9c**	6.85	412.2108	412.2118	−2.43	C_24_H_29_NO_5_	dihydroxylation	-	**+**	-	-	-	-
**M9d**	7.67	412.2108	412.2118	−2.43	C_24_H_29_NO_5_	dihydroxylation	-	-	-	+	-	-
**M9e**	8.06	412.2106	412.2118	−2.91	C_24_H_29_NO_5_	dihydroxylation	-	-	-	-	-	-
**M9f**	8.39	412.2112	412.2118	−1.46	C_24_H_29_NO_5_	dihydroxylation	-	+	-	-	-	-
**M9g**	8.65	412.2109	412.2118	−2.18	C_24_H_29_NO_5_	dihydroxylation	-	+	-	-	-	-
**M9h**	9.09	412.2108	412.2118	−2.43	C_24_H_29_NO_5_	hydroxylation + *N*-oxidation	+	+	-	-	-	-
**M9i**	9.53	412.2110	412.2118	−1.94	C_24_H_29_NO_5_	hydroxylation + *N*-oxidation	-	-	-	-	-	-
**M10a**	3.21	306.1692	306.1700	−2.61	C_17_H_23_NO_4_	hydroxylation + *N*-debenzylation	-	+	+	+	-	-
**M10b**	3.57	306.1692	306.1700	−2.61	C_17_H_23_NO_4_	hydroxylation + *N*-debenzylation	-	+	-	-	-	-
**M10c**	3.65	306.1693	306.1700	−2.29	C_17_H_23_NO_4_	hydroxylation + *N*-debenzylation	-	-	+	+	-	-
**M10d**	4.11	306.1694	306.1700	−1.96	C_17_H_23_NO_4_	hydroxylation + *N*-debenzylation	+	+	+	+	-	+
**M11a**	2.49	292.1536	292.1543	−2.40	C_16_H_21_NO_4_	*O*-demethylation + *N*-debenzylation + hydroxylation	-	-	-	+	-	-
**M11b**	3.21	292.1543	292.1543	0.00	C_16_H_21_NO_4_	*O*-demethylation + *N*-debenzylation + hydroxylation	-	-	-	+	-	-
**M12**	5.26	368.1851	368.1856	−1.36	C_22_C_25_NO_4_	didemethylation + hydroxylation	-	-	+	+	-	-
**M13**	2.56	262.1433	262.1438	−1.91	C_15_H_19_NO_3_	didemethylation + *N*-debenzylation	-	-	+	+	-	-
**M14a**	5.69	398.1954	398.1962	−2.01	C_23_H_27_NO_5_	*O*-demethylation + dihydroxylation	-	-	-	+	-	-
**M14b**	6.10	398.1956	398.1962	−1.51	C_23_H_27_NO_5_	*O*-demethylation + dihydroxylation	-	-	-	-	-	-
**M14c**	6.66	398.1956	398.1962	−1.51	C_23_H_27_NO_5_	*O*-demethylation + dihydroxylation	-	-	-	-	-	-
**M15a**	5.85	542.2377	542.2385	−1.48	C_29_H_35_NO_9_	6-*O*-demethylation + glucuronidation	+	+	-	-	-	+
**M15b**	6.10	542.2379	542.2385	−1.11	C_29_H_35_NO_9_	5-*O*-demethylation + glucuronidation	+	+	-	+	-	-
**M16a**	6.35	572.2478	572.2490	−2.10	C_30_H_37_NO_10_	hydroxylation+ glucuronidation	+	+	-	-	-	-
**M16b**	7.33	572.2481	572.2490	−1.57	C_30_H_37_NO_10_	hydroxylation+ glucuronidation	-	+	-	+	-	-
**M16c**	7.42	572.2483	572.2490	−1.22	C_30_H_37_NO_10_	hydroxylation+ glucuronidation	-	-	-	-	-	-
**M17a**	5.16	528.2222	528.2228	−1.14	C_28_H_33_NO_9_	didemethylation + glucuronidation	+	+	-	-	+	+
**M17b**	5.83	528.2222	528.2228	−1.14	C_28_H_33_NO_9_	didemethylation + glucuronidation	+	+	-	-	-	+
**M18a**	3.52	544.2169	544.2177	−1.47	C_28_H_33_NO_10_	didemethylation + glucuronidation + hydroxylation	-	-	-	-	-	-
**M18b**	4.19	544.2174	544.2177	−0.55	C_28_H_33_NO_10_	didemethylation + glucuronidation + hydroxylation	-	-	-	-	-	-
**M18c**	5.01	544.2173	544.2177	−0.73	C_28_H_33_NO_10_	didemethylation + glucuronidation + hydroxylation	-	-	-	-	-	-
**M18d**	6.05	544.2175	544.2177	−0.37	C_28_H_33_NO_10_	didemethylation + glucuronidation + hydroxylation	-	+	-	-	-	-
**M19a**	4.20	558.2324	558.2334	−1.79	C_29_H_35_NO_10_	*O*-demethylation + hydroxylation + glucuronidation	-	-	-	-	-	-
**M19b**	4.41	558.2324	558.2334	−1.79	C_29_H_35_NO_10_	*O*-demethylation + hydroxylation + glucuronidation	+	+	-	-	-	-
**M19c**	4.88	558.2322	558.2334	−2.15	C_29_H_35_NO_10_	*O*-demethylation + hydroxylation + glucuronidation	-	+	-	-	-	-
**M19d**	5.13	558.2323	558.2334	−1.97	C_29_H_35_NO_10_	*O*-demethylation + hydroxylation + glucuronidation	-	+	-	-	-	-
**M19e**	6.10	558.2324	558.2334	−1.79	C_29_H_35_NO_10_	*O*-demethylation + *N*-oxidation + glucuronidation	-	+	-	-	-	-
**M19f**	6.53	558.2324	558.2334	−1.79	C_29_H_35_NO_10_	*O*-demethylation + *N*-oxidation + glucuronidation	-	+	-	-	-	-
**M19g**	6.81	558.2321	558.2334	−2.33	C_29_H_35_NO_10_	*O*-demethylation + *N*-oxidation + glucuronidation	-	-	-	-	-	-
**M20**	2.02	452.1906	452.1915	−1.99	C_22_H_29_NO_9_	*O*-demethylation + *N*-debenzylation + glucuronidation	+	+	-	-	-	-
**M21a**	8.14	446.1621	446.1632	−2.47	C_23_H_27_NO_6_S	6-*O*-demethylation + sulfation	-	+	-	+	-	-
**M21b**	8.45	446.1621	446.1632	−2.47	C_23_H_27_NO_6_S	5-*O*-demethylation + sulfation	-	+	-	-	-	-
**M22**	7.94	432.1463	432.1475	−2.78	C_22_H_25_NO_6_S	didemethylation + sulfation	-	-	-	+	-	-
**M23a**	6.48	462.1571	462.1581	−2.16	C_23_H_27_NO_7_S	*O*-demethylation + hydroxylation + sulfation	-	-	-	-	-	-
**M23b**	6.72	462.1572	462.1581	−1.95	C_23_H_27_NO_7_S	*O*-demethylation + hydroxylation + sulfation	-	-	-	-	-	-
**M23c**	8.60	462.1575	462.1581	−1.30	C_23_H_27_NO_7_S	*O*-demethylation + hydroxylation + sulfation	-	-	-	+	-	-
**M24**	3.70	356.1155	356.1162	−1.97	C_16_H_21_NO_6_S	*O*-demethylation + *N*-debenzylation + sulfation	-	+	-	-	-	-

^a^ Abbreviations: MB: metabolomics; FBMN: feature-based molecular networking.

## Data Availability

The original contributions presented in this study are included in the article/Supplementary Materials. Further inquiries can be directed to the corresponding author(s).

## Supplementary Materials

The following supporting information can be downloaded at: https://www.mdpi.com/article/10.3390/pharmaceutics17010115/s1, Figure S1: Previously proposed metabolic pathway of donepezil in liver microsomes (M), rats (R) and humans (H), Figure S2: Optimization of extraction solvents for the analysis of donepezil and its four metabolites in rat urine (A), feces (B), and liver (C), Figure S3: Representative extracted ion chromatograms of donepezil (**M0**) and its metabolites obtained from the liquid chromatography–high-resolution mass spectrometric analysis of (A) urine, (B) feces, and (C) liver tissues obtained from rats after oral administration of donepezil (30 mg/kg). NL stands for normalization level, which describes the intensity of the highest peak in each chromatogram, Figure S4: (A) Representative product ion scan mass spectra of donepezil (**M0**) and (B–BL) its metabolites (**M1**–**M24**) obtained from liquid chromatography–high-resolution mass spectrometric analysis of urine, feces, or liver tissues obtained from rats after oral administration of donepezil (30 mg/kg), and their proposed fragmentation schemes. The information on each metabolite is given in Table 1, Figure S5: The fragmentation schemes of dihydroxydonepezil (The structures in square brackets were not observed in MS/MS spectra of dihydroxydonepezil).
