# Supplementary material for: Revisiting the Metabolism of Donepezil in Rats Using Non-Targeted Metabolomics and Molecular Networking
Source: Pharmaceutics. 2025 Jan 15;17(1):115. doi: 10.3390/pharmaceutics17010115 (PMC11769037; doi:10.3390/pharmaceutics17010115)
Supplement: Supplementary file 1 [file pharmaceutics-17-00115-s001.zip › pharmaceutics-3397455-supplementary.pdf]

*Supplementary Materials*

# Revisiting the Metabolism of Donepezil in Rats Using Non-Targeted Metabolomics and Molecular Networking

**Eun-Ji Park, Eui-Hyeon Kim, Ki-Young Kim, Ji-Hyeon Jeon, Im-Sook Song, So-Young Park and Kwang-Hyeon Liu**

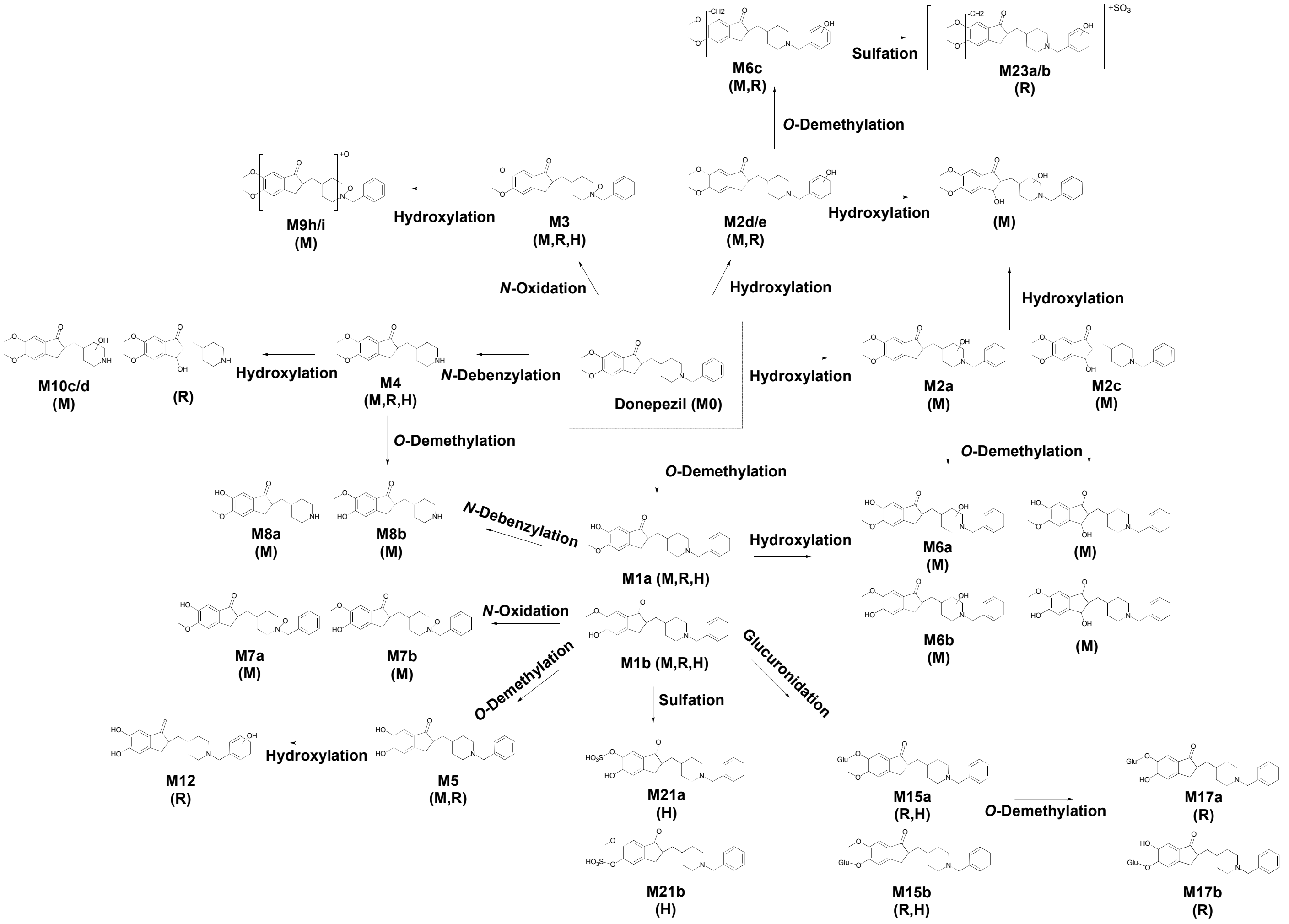


**Figure S1.** Previously proposed metabolic pathway of donepezil in liver microsomes (M), rats (R) and humans (H).

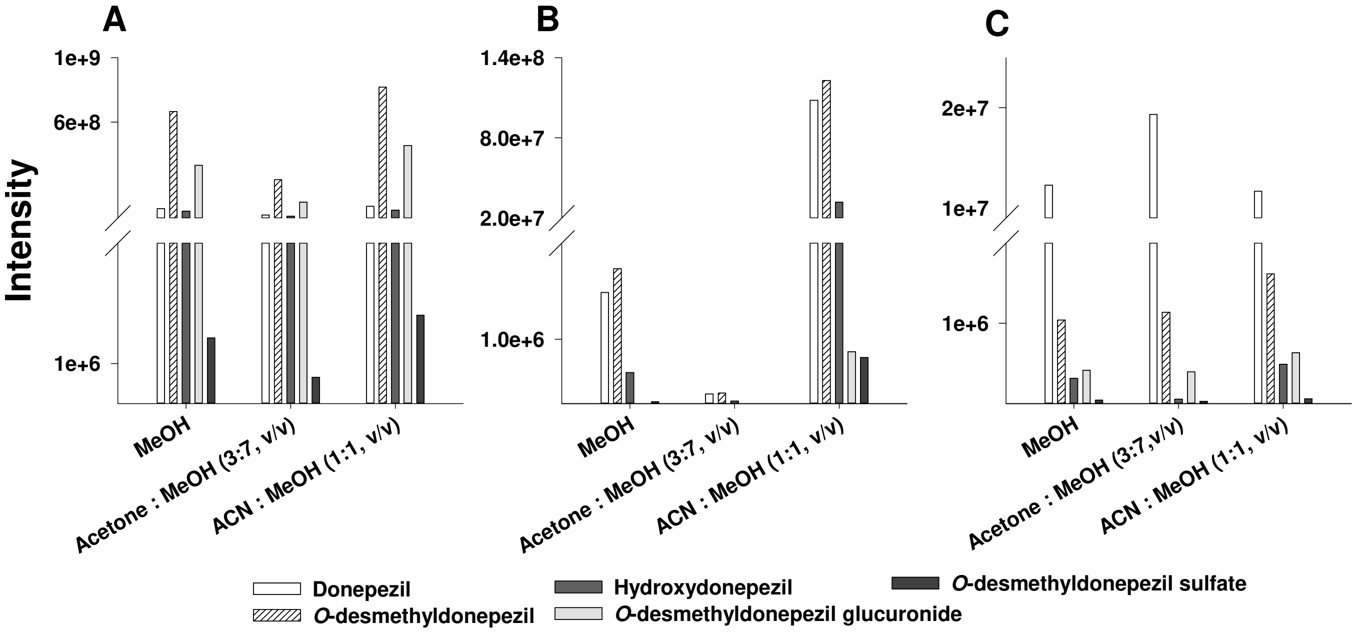


**Figure S2.** Optimization of extraction solvents for the analysis of donepezil and its four metabolites in rat urine (A), feces (B), and liver (C).

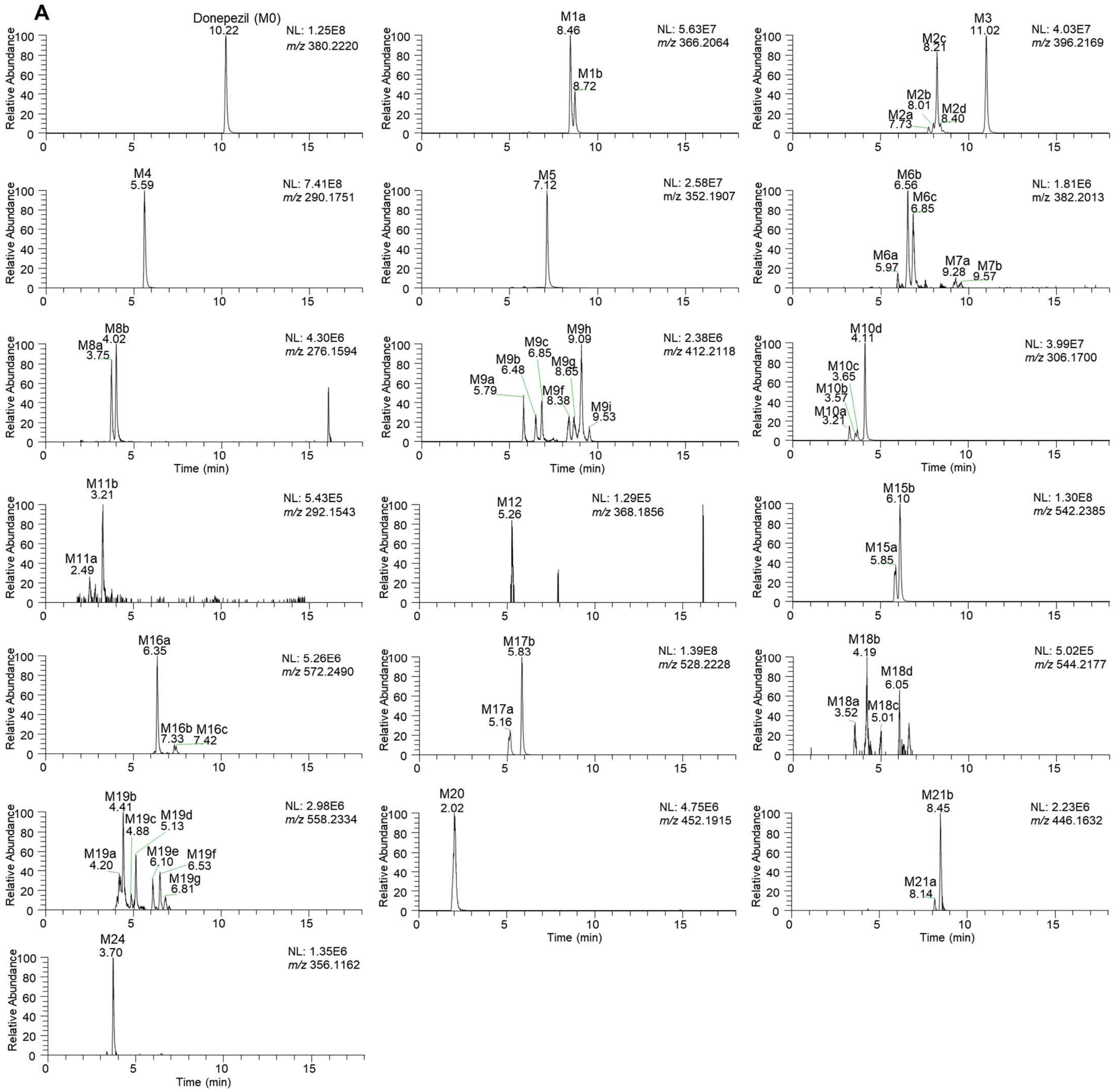


**Figure S3.** *Cont.*

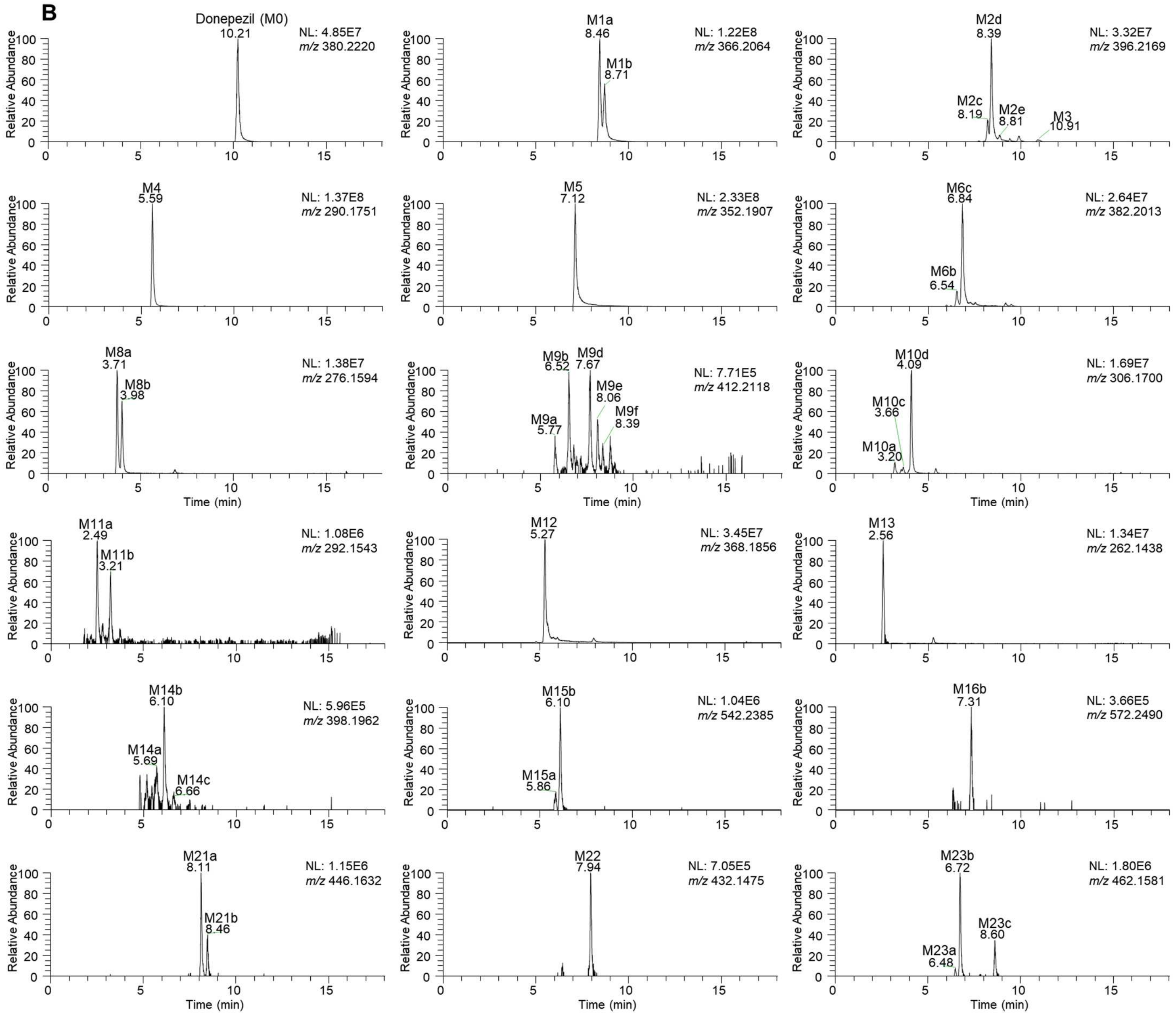


**Figure S3.** *Cont.*

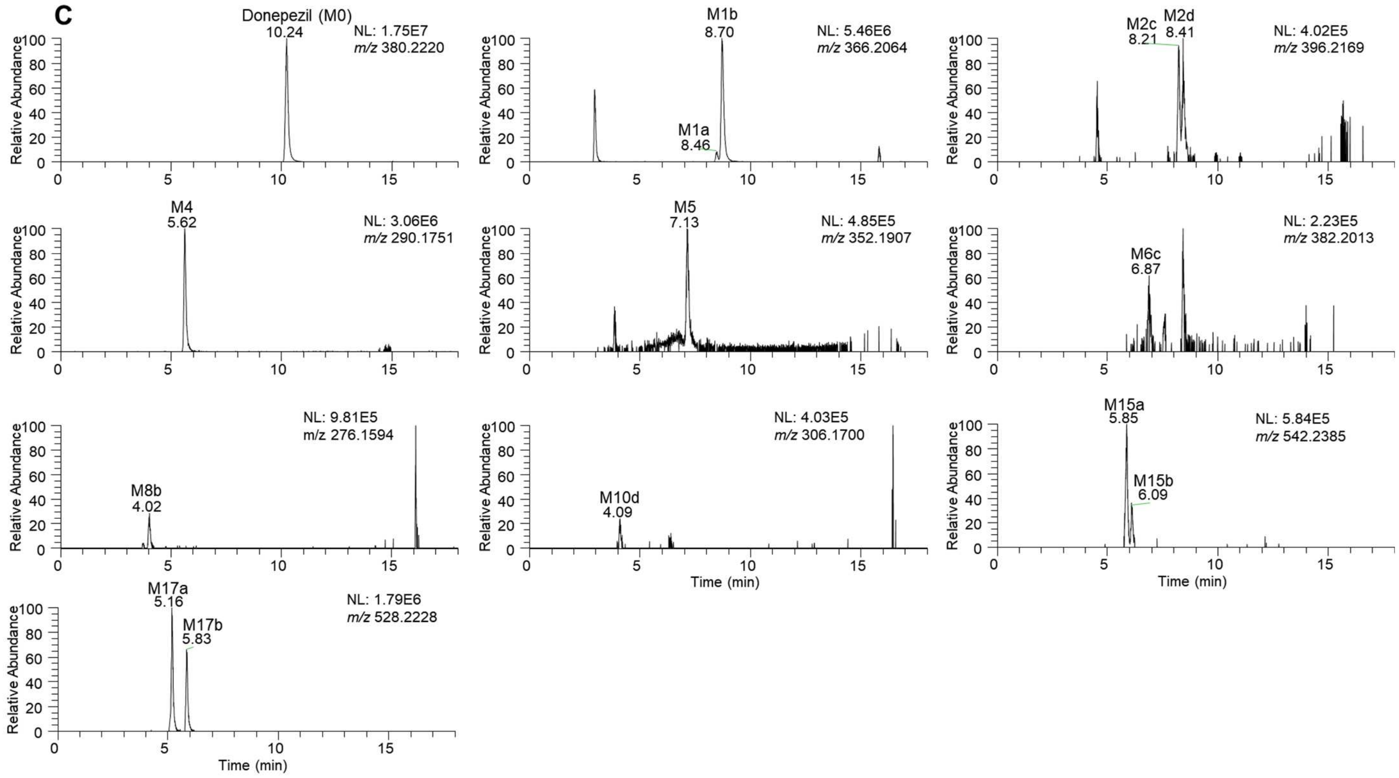


**Figure S3.** Representative extracted ion chromatograms of donepezil (**M0**) and its metabolites obtained from the liquid chromatography–high resolution mass spectrometric analysis of (A) urine, (B) feces, and (C) liver tissues obtained from rats after oral administration of donepezil (30 mg/kg). NL stands for normalization level, which describes the intensity of the highest peak in each chromatogram.

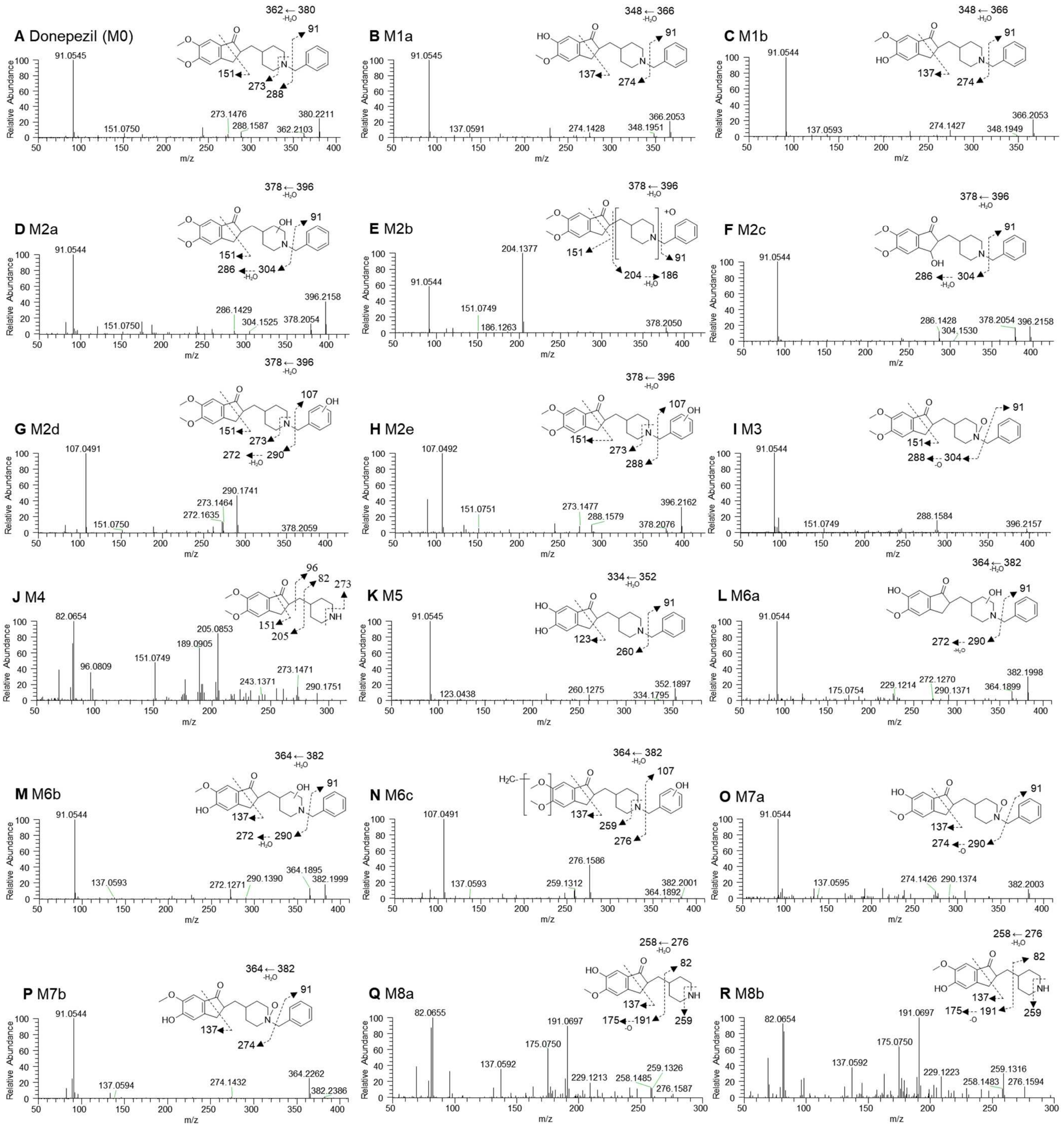


**Figure S4.** *Cont.*

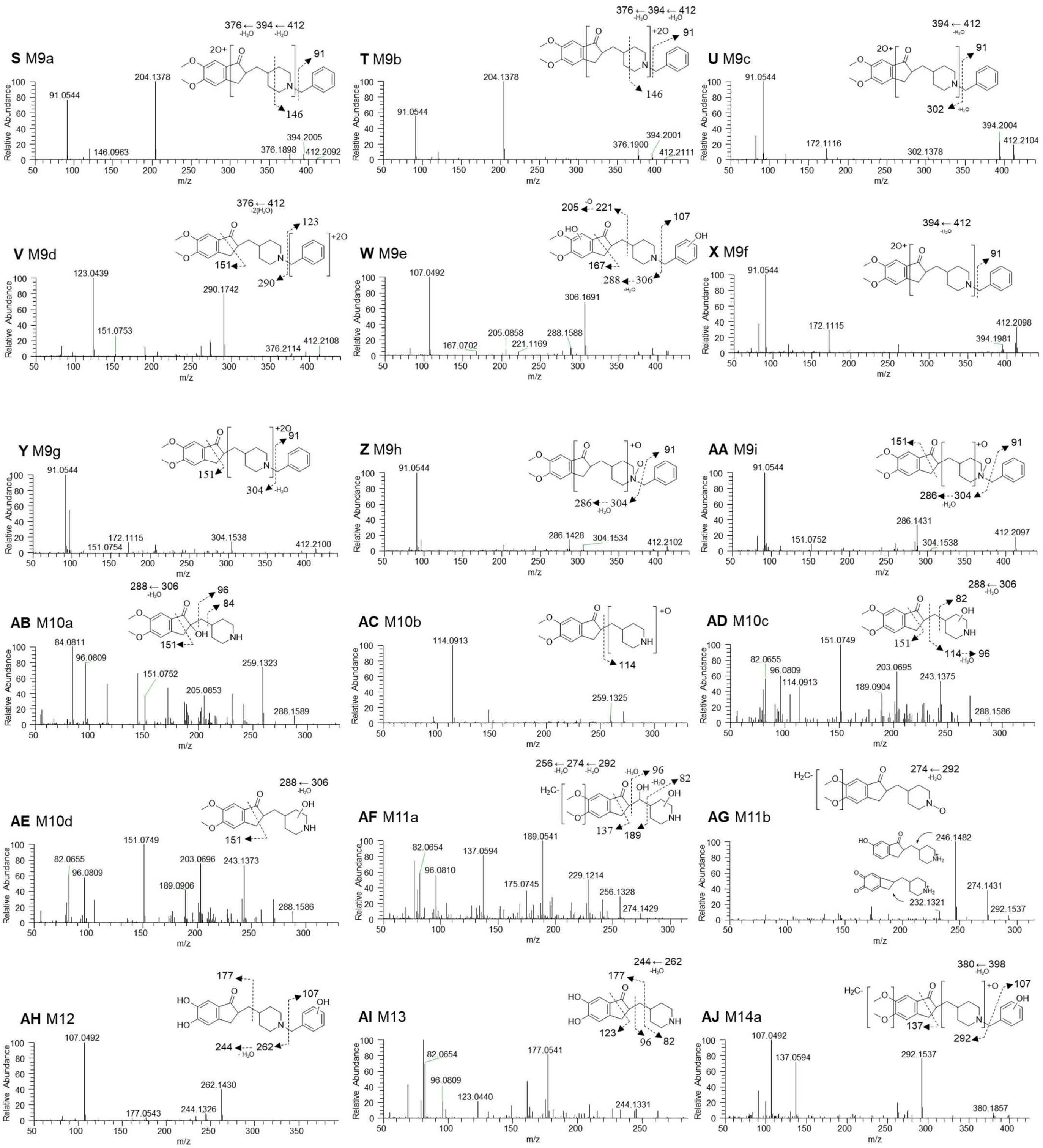


**Figure S4.** *Cont.*

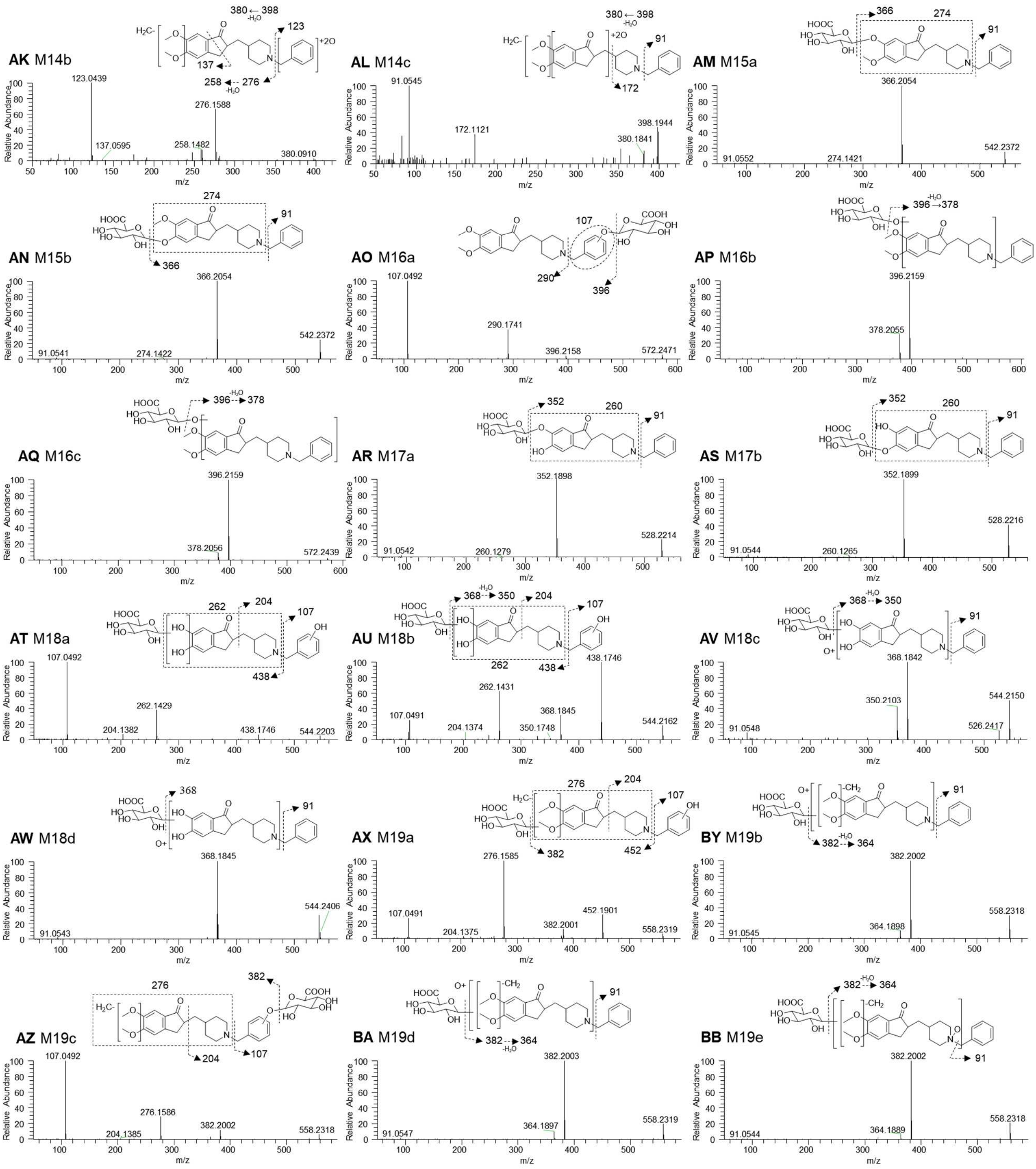

**Figure S4.** *Cont.*

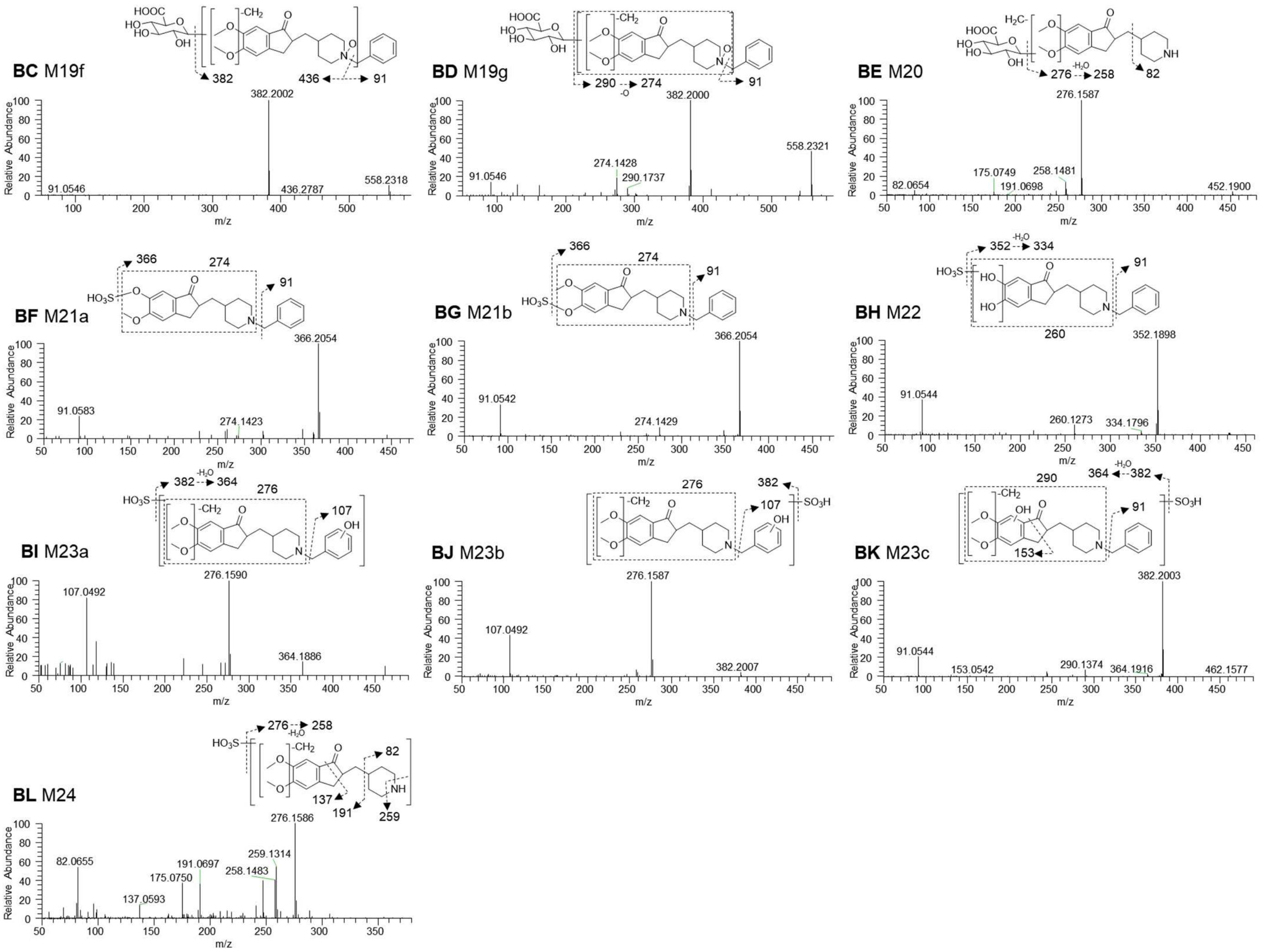


**Figure S4.** (A) Representative product ion scan mass spectra of donepezil (M0) and (B–BL) its metabolites (M1–M24) obtained from liquid chromatography-high resolution mass spectrometric analysis of urine, feces, or liver tissues obtained from rats after oral administration of donepezil (30 mg/kg), and their proposed fragmentation schemes. The information on each metabolite is given in Table 1.

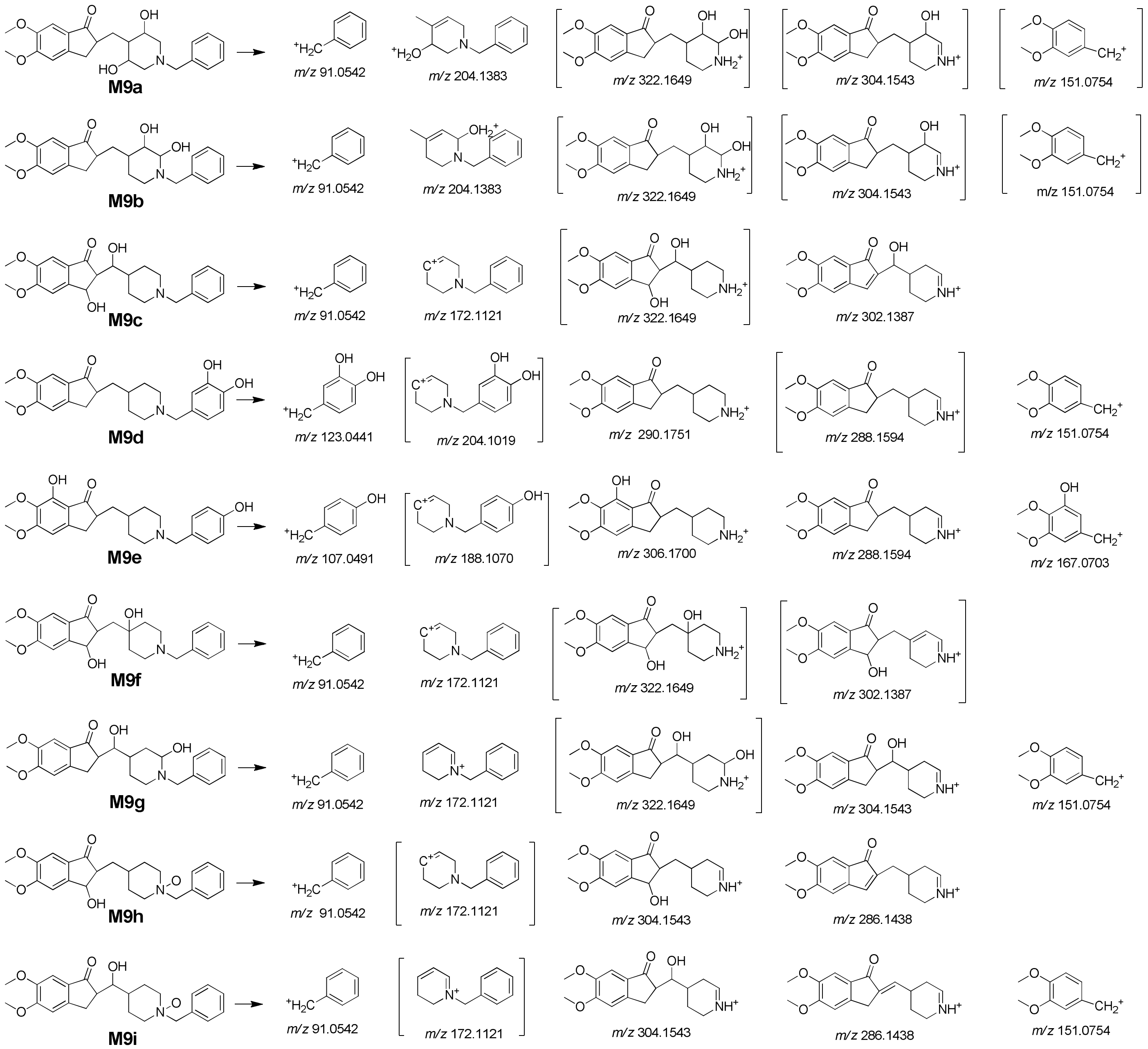


**Figure S5**. The fragmentation schemes of dihydroxydonepezil (The structures in square brackets were not observed in MS/MS spectra of dihydroxydonepezil).
